# Adaptive Immunity in Hypertension

**DOI:** 10.1007/s11906-019-0971-6

**Published:** 2019-07-18

**Authors:** Tomasz P. Mikolajczyk, Tomasz J. Guzik

**Affiliations:** 1 Department of Internal and Agricultural Medicine, Faculty of Medicine, Jagiellonian University Medical College, Krakow, Poland; 2 BHF Centre for Excellence, Institute of Cardiovascular and Medical Sciences, University of Glasgow, Glasgow, UK

**Keywords:** Adaptive immunity, Hypertension, T cell, B cell, Antibody, Cytokine

## Abstract

**Purpose of Review:**

In recent years, a vast body of evidence has accumulated indicating the role of the immune system in the regulation of blood pressure and modulation of hypertensive pathology. Numerous cells of the immune system, both innate and adaptive immunity, have been indicated to play an important role in the development and maintenance of hypertension. The purpose of this review was to summarize the role of adaptive immunity in experimental models of hypertension (genetic, salt-sensitive, and Angiotensin (Ang) II induced) and in human studies. In particular, the role of T and B cells is discussed.

**Recent Findings:**

In response to hypertensive stimuli such as Ang II and high salt, T cells become pro-inflammatory and they infiltrate the brain, blood vessel adventitia and periadventitial fat, heart, and the kidney. Pro-inflammatory T cell–derived cytokines such as IFN-γ and TNF-α (from CD8+ and CD4+Th1) and IL-17A (from the γδ-T cell and CD4+Th17) exacerbate hypertensive responses mediating both endothelial dysfunction and cardiac, renal, and neurodegenerative injury. The modulation of adaptive immune activation in hypertension has been attributed to target organ oxidative stress that leads to the generation of neoantigens, including isolevuglandin-modified proteins. The role of adaptive immunity is sex-specific with much more pronounced mechanisms in males than that in females. Hypertension is also associated with B cell activation and production of autoantibodies (anti-Hsp70, anti-Hsp65, anti-Hsp60, anti-AT1R, anti-α1AR, and anti-β1AR). The hypertensive responses can be inhibited by T regulatory lymphocytes (Tregs) and their anti-inflammatory IL-10.

**Summary:**

Adaptive immunity and its interface with innate mechanisms may represent valuable targets in the modulation of blood pressure, as well as hypertension-related residual risk.

## Introduction

**Fig. 1:**
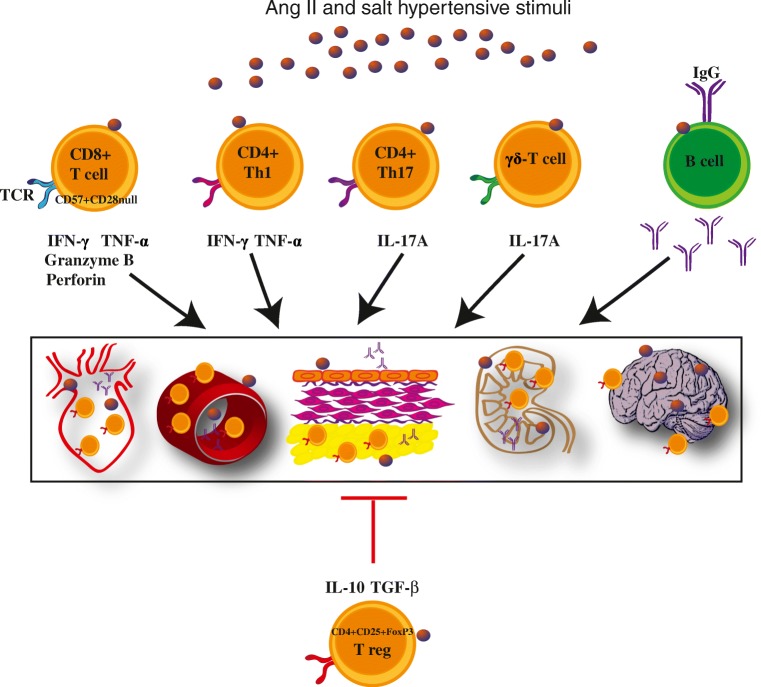
The role of adaptive immunity in the development and maintenance of hypertension. T cells in response to Ang II and/or high-salt stimuli become pro-inflammatory and infiltrate the brain, blood vessels especially adventitia and periadventitial fat, heart, and kidney. T cells produce pro-inflammatory cytokines such as IFN-γ and TNF-α (CD8+, CD4+Th1) and IL-17A (γδ-T cell, CD4+Th17), which exacerbate hypertensive responses and induce endothelial dysfunction as well as cardiac, renal, and neurodegenerative injury. In hypertension, B cell and their antibodies play the role in end-organ damage. The hypertensive responses are inhibited by T regulatory cells (Treg) and their anti-inflammatory IL-10

In recent years, accumulating evidence indicates the role of the immune system in the regulation of blood pressure and cardiovascular risk linked to hypertension. While our initial studies using RAG1^−/−^ mice have shown the pathogenetic role of T cells in this process, subsequent cooperation of numerous cells of the immune system, both innate and adaptive immunity, has been implicated in the development and maintenance of hypertension (Fig. 1) [1••, 2–7]. The first line of defense includes the innate response and takes place relatively very fast. The second line of the defense, namely adaptive immunity, is characterized by a delayed but very targeted response. In terms of the development of hypertension, the interaction between these two components of the immune system seems to be essential [8, 9••].

### Innate-Adaptive Immunity Interface in Initiation of Inflammation

Innate cells, such as granulocytes, monocytes, macrophages, and dendritic cells, express the pathogen recognition receptors (PRRs; such as Toll-like receptors (TLRs)), and they can recognize pathogen-associated molecular patterns (PAMPs) or damage-associated molecular patterns (DAMPs). A number of molecules, of importance to hypertension, may act as DAMPs activating Toll-like receptors. These include nuclear or cytosolic proteins as well as neoantigens [10]. The latter represent forms of “new antigens” arise in the stress condition, and they are generated, for example in the context of hypertension, during oxidative stress or they are then released from injured tissue [11]. These molecules, acting as DAMPS, may activate innate immunity, mainly through interaction with Toll-like receptors (TLRs) or may also be presented by antigen-presenting cells (APC) in the context of major histocompatibility complex II (MHC II) initiating adaptive immunity leading to the activation of T and B lymphocytes [12, 13••]. In classical immunology, the key role of the adaptive immunity is to create memory cells that recognize these specific antigens during the re-appearance in the environment [3, 14, 15]. In the future, due to the presence of memory T cells, the response is faster and more effective [16]. In hypertension, indeed, the accumulation of memory cells has been described in both animal models [17] and humans [3].

### Role of T Cells and Their Subsets in Experimental Model of Hypertension

In many experimental models of hypertension including genetic model and salt or angiotensin (Ang II)-induced model, the key role of T cells has been demonstrated [1••, 2, 3].

Initial reports used a genetic model of essential hypertension and revealed that spontaneously hypertensive rats (SHRs) had reduced numbers of T cells in the thymus and that restoring thymic function by histocompatible thymus grafts or thymic extracts suppressed the development of hypertension [18–20]. The immunological restoration was associated with significant suppression of high blood pressure. Depression of T cell function was accompanied by an appearance of natural thymocytotoxic autoantibody (NTA) that was decreased after thymus grafts or extracts [20]. Further studies demonstrated that T cells and especially non-helper subsets were depressed in both the prehypertensive and developmental phases of hypertension in SHR [21]. Subsequent studies performed by Rodriguez-Iturbe et al. showed that lymphocytes and macrophage infiltrate the kidney of SHR and that this phenomenon is more pronounced with age. The interventions leading to the reduction of those cells were associated with improvement of hypertension. Six-month-old SHR showed increased content of T helper cells and high CD4/CD8 ratio [22]. Interestingly, hypertensive responses depend on the sex of the animals and this impacts the T cell profile [23]. Moreover, distinct SHR lines differ in their susceptibility to hypertensive end-organ disease [24]. SHR-A3 is end-organ injury susceptible line whereas SHR-B2 is not [24–26]. This phenomenon is associated with the regulation of Tregs [24–26]. SHR-A3 has an additional hypertension locus that contributes to higher blood pressure increase [24]. Moreover, hypertension can be modified in genetically hypertensive rats by immunosuppressive therapy using mycophenolate mofetil (MMF). After the treatment with MMF, the systemic hypertension was blunted which was accompanied by lymphocyte, macrophage, and angiotensin II–positive cell reductions in the kidney. This effect was accompanied by reduced oxidative stress [27]. Mycophenolate mofetil also prevented salt-sensitive hypertension (SSHTN) in Sprague Dawley rats receiving Ang II. In this model, kidney injury was significantly reduced by MMF administration. This was accompanied by decreased proliferation, T cell infiltration, and activation [28•]. In Dahl salt-sensitive (SS) rats, increasing salt consumption enlarged renal infiltration of T lymphocytes and increased arterial blood pressure and albumin excretion and resulted in renal glomerular and tubular damage [29]. Infiltrating T cells produced Ang II that caused renal disease. Suppression of T cell decreased intrarenal Ang II and prevented Dahl SS hypertension [29]. Interestingly, the high-protein diet was associated with greater numbers of infiltrating T lymphocytes in kidney and highest mean arterial blood pressure and urine to creatinine ratio in comparison with a low-protein diet [30]. This observation shows that hypertension in Dahl SS rats is sensitive to both NaCl and protein intake [30]. Viel et al. have demonstrated that T lymphocyte contribution to vascular inflammation is related to the influence of chromosome 2 in genetic salt-sensitive hypertension [31]. SSBN2 rats in which chromosome 2 has been transferred from normotensive to hypertensive animals had reduced systolic blood pressure compared with Dahl rats. Hypertensive rats exhibited increased inflammatory markers and mediators especially VCAM-1, ICAM-1, CCR5, and CD4 compared with normotensive Brown Norway rats. SSBN2 rats revealed a reduction in all these markers in comparison with Dahl rats. Aortic expression of Foxp3 and transforming growth factor beta (TGF-beta) and IL-10 were enhanced in SSBN2 rats indicating that Tregs attenuate salt-sensitive hypertension [31]. While all these studies were very informative, the direct proof of involvement of T cells in hypertension was provided by our studies in mice lacking recombination activating gene 1 (RAG1^−/−^), which was then confirmed in RAG1^−/−^ Dahl salt-sensitive rats [1••, 32]. Those animals lack mature T and B cells in the circulation and in the spleen, creating a unique opportunity to address the role of these cells in hypertension. RAG1^−/−^ mice have blunted hypertension and did not develop vascular pathologies during Ang II or deoxycorticosterone acetate (DOCA)-salt-induced hypertension. However, the adoptive transfer of T cells but not B lymphocytes restored these abnormalities [1••]. The infiltration of T cells into the kidney upon high-salt intake was blunted in RAG1^−/−^ rats compared with the controls. This phenomenon was accompanied by lower arterial blood pressure and urinary albumin excretion [32]. Further evidence for the role of the adaptive immunity in hypertension comes from the study of Crowley et al. [33]. They demonstrated that SCID (severe combined immunodeficiency) mouse strain lacking in T and B lymphocytes is protected against Ang II–dependent hypertension. SCID mice developed less cardiac hypertrophy and had significant reductions in heart and kidney injury following Ang II challenge. This phenomenon was associated with the stimulation of eNOS- and COX-2-dependent pathways [33]. In Ang II hypertensive rats, Shao et al. have demonstrated T helper lymphocytes imbalance with an increase of Th1 and a decrease of Th2 in the spleen and kidney. The administration of Ang II receptor blocker (ARB) olmesartan, but not vessel dilatator hydralazine, ameliorated the manifestations of disease and the imbalance in T helper subsets [34]. Further mechanisms were provided by an interesting study by Trott et al. who have shown that CD8+ T cells are the primarily activated T lymphocytes in hypertension and CD8^−/−^ mice were protected from angiotensin-induced endothelial dysfunction and vascular remodeling in the kidney [35]. Also, double-negative T (DN-T) cells (CD3+CD4−CD8−) are abundantly recruited to the vasculature especially perivascular adipose tissue in Ang II–dependent hypertension [1••, 2]. It is interesting to note that the loss of lymphocyte adaptor protein LNK (also known as SH2B3) exacerbates inflammation and renal and vascular dysfunction following angiotensin II–induced hypertension [36]. Recently, we provided the evidence that regulated on activation, normal T cell expressed and secreted (RANTES) chemokine is essential for T cell homing in hypertension. Angiotensin II–induced hypertension was associated with an increase of RANTES level in perivascular adipose tissue (PVAT). Also, the expression of CCR1, CCR3, and CCR5 was higher in T cells infiltrating PVAT upon Ang II infusion. Moreover, RANTES^−/−^ protects against vascular leukocyte and especially T lymphocyte infiltration, endothelial dysfunction, and oxidative stress [2]. Interestingly, Itani et al. have used a humanized mouse model in which the murine immune system was replaced by the human immune cells. They observed increased infiltration of human leukocytes, T cells, and especially CD4+ subsets in thoracic lymph nodes, thoracic aorta, and kidney in response to Ang II infusion. Also, CD8+ infiltration was higher in both lymph nodes and thoracic aorta in hypertensive animals compared with normotensive. The increase in memory T cells CD3+CD45RO+ was noted in the aortas and lymph nodes. In this model, human T cells become activated and invade end-organ tissue, in response to Angiotensin II stimuli [3].

The mechanisms of activation of adaptive immunity in hypertension remain not fully defined. The role of the central nervous system (CNS) in this process has been compellingly demonstrated. It has been shown that the elevation in blood pressure was eliminated by anteroventral third cerebral ventricle (AV3V) lesions in mice. Also, the activation of circulating T cells and the vascular infiltration of leukocytes were reduced in this model [37]. Recently, Carnevale et al. have demonstrated that splenectomized mice are protected from Ang II–induced blood pressure increase. They found that splenic placental growth factor (PIGF) is indispensable for the onset of hypertension. The role of PIGF is repressing of the expression of protein Timp3 (tissue inhibitor of metalloproteinases 3) involving the transcriptional Sirt1-p53 axis. PIGF is essential for T cell co-stimulation via CD86. As a result, the deployment of those cells towards vasculature and target organs is observed [38•]. Splenectomized mice or mice with left coeliac ganglionectomy were also unable to increase blood pressure upon DOCA-salt challenge. The intact neurosplenic sympathetic drive resulted in increased PIGF expression in DOCA-salt mice. PIGF ^−/−^ mice subjected to DOCA-salt model were protected from increased blood pressure and T cell co-stimulation and deployment towards kidney [39]. Chronic high blood pressure is also associated with brain damage and the development of neurodegenerative injury in hypertensive individuals [40].

Seminal work by Kirabo et al. linked hypertensive oxidative stress to the activation of adaptive immunity [13••]. It has been well defined that hypertensive responses are also associated with an increase in oxidative stress [1••, 2, 41]. Superoxide production is increased due to increased NOX activity, and this effect is associated with reduced NO production and impaired endothelium–dependent vasodilatation [42]. Hypertension-induced oxidative stress can also affect vascular smooth muscle cell function, which plays an important role in contractility [43]. It has been shown that the pro-inflammatory phenotype of SMC was essential to the development of pulmonary hypertension [44]. Moreover, oxidative stress can affect the proteins, which are next internalized by antigen-presenting cells (APC). In multiple models of hypertension, Kirabo et al. demonstrated that the proteins oxidatively modified by highly reactive gamma-ketoaldehydes (isoketals/isolevuglandins) are formed and finally they are presented by dendritic cells to T cells. After this contact, T cells become activated and pro-inflammatory [13••]. Interestingly, the scavenging of isolevuglandins prevented experimental hypertension and may be the potential useful treatment strategy for this disease [13••]. T cell activation requires receptor ligation and co-stimulation. The second signal is supplied by the interaction between CD80 and CD86 (B7.1 and B7.2, respectively) on APC with co-receptor CD28 on T cells. Ang II–dependent hypertension increased the content of CD86+ dendritic cells. Interestingly, the blockade of B7-dependent co-stimulation with CTLA-4-Ig reduced DOCA-salt and Ang II–induced hypertension [45•]. CTLA-4-Ig abrogated activation of circulating T cells, cytokine production, and their vascular accumulation [45•]. Deficiency in B7 ligands was accompanied by minimal blood pressure elevation and vascular inflammation despite Ang II infusion [45•]. Itani and colleagues have shown that the formation of effector memory T (T(EM)) cells requires the co-stimulatory molecule CD70 on APC [17]. During a murine N(omega)-nitro-L-arginine methyl ester hydrochloride (L-NAME)/high-salt model of hypertension, T(EM) accumulated in the kidney and produced IFN-γ and IL-17A. This phenomenon is accompanied by an increased expression of CD70 on macrophages and dendritic cells. CD70 deficiency led to a lack of T(EM) accumulation and resulted in no development of hypertension and renal damage [17]. The ablation of myeloid CD11c+APC, participated in renal sodium transport and prevented cardiac hypertrophy and the induction of several indicators of renal and cardiac damage in response to angiotensin II and high-salt diet [12]. APC were necessary for the induction of intrarenal renin–angiotensin system components and affected the modulation of natriuresis and tubular sodium transporters [12]. The data provided by Shah et al. revealed that myeloid-derived suppressor cells (MDSCs) play an important role in attenuating hypertensive responses in different models including Ang II, L-NG-nitroarginine methyl ester, and high salt. The depletion of MDSCs increased blood pressure and renal inflammation [46]. Myeloid-derived suppressor cells have an expression of markers and transcription factors, which are associated with immunosuppression and immaturity. Suppression of T cell activation was dependent on hydrogen peroxide produced by MDSCs [46]. Recently, we have shown that during Ang II–dependent hypertension, the Sphingosine kinase 1 (Sphk1) was upregulated in the vasculature. This is important, taking into account the role of S1P (sphingosine-1-phosphate) in the regulation of immune cell trafficking. Chronic infusion (S1P) resulted in increased vasoconstriction and endothelial dysfunction. Ang II–induced hypertension was blunted in Sphk1^−/−^ mice. In those animals, decreased vasoconstriction was observed and enhanced endothelial dysfunction suggesting the protective role of Sphk1 in the endothelium [47].

### Role of T Cells in Hypertension in Humans

Interestingly, the analysis of the T cell population from hypertensive individuals revealed an increased percentage of immunosenescence, pro-inflammatory, and cytotoxic CD8+ cells. These cells were characterized by the expression of CD57 antigens and lack of expression of CD28 molecules (CD8+CD57+CD28null). CD8+ cells derived from patients with hypertension produced a higher level of perforin and granzyme B in comparison with normotensive individuals. Furthermore, hypertension was associated with a higher percentage of CD8+ T cells producing both IFN-γ and TNF-α. This effect was not observed in CD4+ T cells but in those cells a higher percentage of perforin-positive cells was noticed in hypertensive subjects in comparison with normotensive individuals. Renal tissue biopsies confirmed higher infiltration of T cells and their subsets CD4+ and CD8+ in patients with hypertensive nephrosclerosis [48]. Interestingly, this phenomenon was accompanied by an increased level of CHCR3 chemokines (monokine induced by γ IFN (MIG), IFN-γ-induced protein 10 (IP-10), and interferon-inducible T cell alpha chemoattractant (I-TAC)), which are known to play a role in pro-inflammatory T cell tissue homing [48]. Immunosenescence T cells appear as a consequence of repeated antigenic stimulation, and they are associated with age [11]. In hypertensive individuals, also the increase percentage of CD45RO both CD4+ and CD8+ T cell subsets was observed [3]. This finding of increased circulating memory T cells provides evidence of the activation of the immune system and might have pathophysiological significance for sustaining hypertension in humans. This observation was also accompanied by an elevated level of RANTES chemokine in the plasma. In a human cohort of subjects with metabolic syndrome and other risk factors for coronary disease, we observed a significant inverse correlation between circulating RANTES level and vascular function measured as flow-mediated dilatation (FMD). In line with this, we found the positive correlation between the biochemical marker for endothelial dysfunction, von Willebrand factor (vWF), and RANTES level [2].

### Sex Differences in T Cells–Mediated Hypertension

The majority of studies regarding the role of T cells in the pathogenesis of hypertension have been performed in male mice. However, both men and women develop hypertension. There is increasing evidence that although T cells also mediate blood pressure development in females, there are differences in T cell profile [49]. Ji et al. have suggested that the progression of renal disease and end-organ damage is faster in men compared with women [50]. In this study, the authors showed that the markers of renal injury, including the glomerulosclerosis index, mean glomerular volume, and proteinuria, were greater in the renal wrap model of hypertension in males compared with females. Also, endothelial NO synthase expression was elevated in males and no differences were noted in eNOS level in females with hypertension. Interestingly, the eNOS was significantly decreased in male medulla but not in the cortex and no differences were observed in females in both renal compartments. Despite the similar degree of hypertension in both males and females, the renal function was dependent on gender [50]. In another study, it has been shown using radio-telemetry that male SHRs have a greater blood pressure than female [51]. The gonadectomy decreased blood pressure in males and had no effect on blood pressure in females. Greater plasma Ang II level was observed in females and similar levels of renal cortical Ang II in comparison with males. Interestingly, females had lower expression of AT(1) receptor in the renal cortex, decreased macrophage infiltration, and decreased oxidative stress which was consistent with lower blood pressure [51]. This is interesting because women more often than men develop inflammatory and autoimmune disorders (e.g., rheumatoid arthritis (RA) and systemic lupus erythematous (SLE)), which are associated with an increased risk of cardiovascular diseases [52–54]. Additionally, in the pathogenesis of preeclampsia, the immune system plays an important role. Especially, this disorder is associated with an increase in Th17 lymphocytes, shift towards Th1 responses, and a decrease in Treg activity both in humans and in the rat model of preeclampsia [55, 56]. Tipton et al. have demonstrated that spontaneously hypertensive female rats had greater renal anti-inflammatory T lymphocyte infiltration than males who had greater CD4+ and Th17 infiltration [23]. Males revealed higher blood pressure than females, who had greater sensitivity to mycophenolate mofetil–induced decrease in lymphocyte counts [23]. Interestingly, the administration of hydrochlorothiazide and reserpine decreased T regulatory cells content only in female SHR abolishing sex differences. This treatment had minimal impact on Th17 cells. To assess the impact of sex hormones to renal immune profile, the gonadectomy was performed. Increased pro-inflammatory markers were observed after gonadectomy providing the suggestion that hormones play anti-inflammatory roles in both males and females [57•]. Brinson et al. have demonstrated that the administration of the nitric oxide synthase (NOS) inhibitor (L-NAME) increases blood pressure in both sexes; however, females exhibited a greater increase in BP than males. L-NAME-induced hypertension was associated with increased renal T cell infiltration with a greater increase in Th17 cells and a greater decrease in Tregs in female SHR in comparison with male. Interestingly, anti-hypertensive therapy significantly reduced the L-NAME-induced increase in renal T cell infiltration in both sexes. This data suggests that NOS is essential in females SHR to maintain BP and limit a pro-inflammatory renal T cell profile in comparison with males [58]. Tregs can be responsible for lower blood pressure in females in comparison with males. Female mice are also protected against high-fat-diet-induced metabolic syndrome. There was an increase in regulatory T cell population in adipose tissue in response to weight gain in females, which was opposite to the phenomenon observed in males. High-fat-diet-fed males developed in this time adipose tissue inflammation, glucose intolerance, and hyperinsulinemia [59].

Ji et al. have provided evidence that the signaling of pro- and anti-inflammatory cytokines differ between the sexes [60]. Sex differences in mean arterial pressure observed in wild type mice, with higher pressure in males, were lost in both sexes in RAG1^−/−^ mice. Blood pressure was higher after the adoptive transfer of male CD3 in comparison with female CD3 into male RAG1^−/−^. RAG1^−/−^ male with male CD3 had a higher percentage of splenic IL-17A and TNF-α T cells and lower plasma of IL-10. RAG1^−/−^ male with female CD3 revealed higher activation and Th1 response in renal inflammation. Greater T cell infiltration into perivascular adipose tissue and kidney was associated with male but not female T cell donor [60]. Pollow et al. using the same model of Ang II–infused RAG1^−/−^ mice demonstrated that systolic blood pressure responses were similar between sexes. Adoptive transfer of male T cells was associated with higher blood pressure in males when compared with females following Ang II infusion. Renal T cell infiltration was significantly increased in males compared with females in control and Ang II–infused mice. This study provided evidence that female RAG1^−/−^ animals are protected from male T cell–mediated increases in hypertension when compared with male knockouts.

Sex difference involves the infiltration of T cell especially in both the kidney and the brain [61]. Recently, Sandberg et al. have shown that CD4+ and CD8+ T cell subsets are different in males and females and sex-specific effects of these cells determine the magnitude of Ang II–induced hypertension [62]. Interestingly, the adoptive transfer of both male CD4+ and CD8+ to male RAG1^−/−^ resulted in greater mean arterial pressure in response to Ang II compared with females derived both CD4+ and CD8+ T cells [62]. Female CD8+ T cells attenuated Ang II–induced hypertension compared with male RAG1^−/−^ mice. Only animals receiving male T cells exhibited T cell infiltration into the subfornical organ. This is consistent with the findings that 17β-estradiol acting via ERs in the SFO inhibiting inflammatory process induced by Ang II especially ROS production [63, 64].

The infiltration of renal FoxP3+CD4+CD3+ T cells was higher in female SHR than that in male in 13-week-old animals. This effect was accompanied by a decreased frequency of ROR-γ+CD4+CD3+ cells. Interestingly, no differences between females and males were noted in 5 weeks of age [65].

Schneider et al. demonstrated that sex could be associated with the degree to which HLA propagate the selection and further expansion of T cells characterizing T cell receptor variable beta chain (TCRBV). Interestingly, immunosequencing of 824 individuals revealed that HLA-associated shaping of TCRBV differed between sexes. CD8 T cells in men with autoimmune disease were capable to expand even without expressing TCRBV with similarity in pivotal HLA-binding regions [66].

### Role of B Cells and Their Antibodies in Hypertension

As a concept of the central role of T cell in hypertension is widely accepted, the role of B cells is not as evident. However, Chan and then subsequently Dingwell provided compelling evidence for the B lymphocyte role in hypertension. Ang II–dependent hypertensive responses were attenuated in B cell-activating factor receptor-deficient (BAFF-R^−/−^) mice, lacking mature B cells. Interestingly, the transfer of B cells to those animals restored blood pressure increase. BAFF-R ^−/−^ was associated with reduced IgG accumulation and macrophage content in the aorta. Those animals were also protected from collagen deposition and aortic stiffening induced by Ang II. The protection from hypertension was also conveyed by the administration of anti-CD20 antibody providing proof of concept for future therapeutic use [67]. Dingwell et al. confirmed that B cell deficiency resulted in lower blood pressure in mice [68]. Mice homozygous for proto-oncogene c-myb had reduced B220+ B cells in peripheral blood and kidney and revealed decreased both systolic and diastolic blood pressure compared with WT animals. Interestingly, those animals had lower susceptibility to DOCA-salt experimental hypertension. Reconstitution of WT mice with bone marrow transplant lacking B cells resulted in decreased blood pressure [68]. This effect was associated with reduced vasopressin receptor 2 (V2R) in the kidney [68].

These data correspond to findings of the role of elevated autoantibody levels in both experimental models of hypertension and in humans. Wu et al. demonstrated that there is an association of antibodies against heat shock protein 70 (Hsp70) with hypertension. The presence of antibodies to Hsp70 was higher in subjects with a blood pressure of 160/95 mmHg than that in those with a blood pressure of 140/90 mmHg [69]. In another study, it has been shown that the level of antibodies against Hsp70 and Hsp65 is elevated in hypertension in comparison with normotensive individuals but no changes in Hsp60, Hsp70, and anti-Hsp60 levels were observed [70]. However, the study performed in patients with coronary heart disease revealed that high concentration of anti-Hsp60 antibodies was elevated in comparison with the controls. Increasing concentration of anti-Hsp60 was associated with a higher risk of CHD, hypertension, and diabetes [71]. Autoantibodies against angiotensin II receptor type 1 (AT1) have been discussed in relation to their role in hypertension for a long time. Their increased levels were found in patients with preeclampsia [72]. Levels of anti-AT1 correlated with the severity of this disease [73]. Also, in malignant hypertension, there was an increased level of autoantibodies against AT1 but not AT2 receptors [74]. Zhu et al. have showed that autoantibodies against AT1 in patients with essential hypertension correlated with the polymorphism of HLA-DRB1 [75]. Liao et al. have demonstrated that in patients with refractory hypertension there is a higher frequency of autoantibodies against AT1-receptor and alpha1-adrenergic receptor (α1-AR) [76]. The elevated level of antibodies against α1-AR in both primary and malignant hypertension has been confirmed in other studies [77, 78]. Immunoadsorption of α1-AR antibodies resulted in blood pressure reduction in patients with refractory hypertension. These antibodies induced signaling pathways involving protein kinase C alpha activation and ERK1/2 phosphorylation, which are important for hypertension and cardiac remodeling [79]. The presence of autoantibodies has been also observed in sera of spontaneously hypertensive rats [80]. Interestingly, sera of SHR contained autoantibodies against both beta 1-adrenoceptor and arterial antigens indicating B lymphocyte involvement in a genetic model of essential hypertension [80, 81]. The role of such antibodies may also be important for target organ damage. Autoimmune reactions to cardiac beta1-adrenergic receptor plays a causal role in dilated cardiomyopathy in an animal model [82]. Interestingly, in patients with hypertension, autoantibodies against cardiovascular L-type Ca2+ channels have been commonly found, especially in patients with comorbidities such as coronary heart disease and left ventricular diastolic dysfunction [83].

### Role of Cytokines Involved in Adaptive Immunity in Hypertension

T cell–derived cytokines play a central role in the pathophysiology of cardiovascular disease and hypertension and contribute to the end-organ damage [1••, 7, 84]. One of the first identified and best-characterized cytokines in relation to hypertension is IL-17. T helper 17 (Th17) cells and their pro-inflammatory cytokine IL-17 play an essential role in hypertensive autoimmune diseases and endothelial dysfunction [85, 86•]. IL-17 increases blood pressure and decreases NO-dependent relaxation responses by activation of RhoA/Rho-kinase. Moreover, the inhibition of Rho-kinase prevented hypertension caused by IL-17 [85]. Interestingly, the vessels from IL-17^−/−^ mice displayed preserved vascular function and decreased superoxide anion production. This effect was accompanied by a significant reduction in T cell infiltration in response to chronic Ang II infusion [86•]. IL-17 together with TNF modulated the expression of over 30 genes including inflammatory cytokines in human aortic smooth muscle cells [86•]. The murine and human Th17 is induced by sodium chloride in vivo. High-salt concentrations activate the p38/MAPK pathway involving a nuclear factor of activated T cells 5 (NFAT5) and serum/glucocorticoid-regulated kinase 1 (SGK1) [87]. This kinase is critical for regulating IL-23R expression by deactivating a direct repressor of this receptor (Foxo1) and stabilizing Th17 phenotype [88••]. Modest increase in salt concentration induces SGK1 expression, promotes IL-23R expression, and enhances Th17 differentiation [88••]. Ang II–dependent hypertension was associated with an increased percentage of both IL17+CD4+ T cells and IL17+CD3+CD4−CD8− T cells in perivascular adipose tissue. The main producers of IL-17 were CD3+CD4−CD8− T lymphocytes [2, 41]. Recently, Saleh et al. provided the evidence that the primary T cell subsets producing IL-17A in the kidney and aorta are γδ T cells and CD4+ T helper 17 cells. Additionally, they found that antibodies against IL-17A or the IL-17 receptor A subunit (IL-17RA) lowered blood pressure and attenuated renal and vascular lymphocyte infiltration in Ang II–infused animals. This phenomenon was not observed when IL-17F antibodies were administered. Interestingly, IL-17A or IL-17RA antibodies reduced transforming growth factor beta (TGF- β) levels compared with control IgG1 antibodies [89]. IL-17A regulates renal sodium transporters [90, 91]. During Ang II–dependent hypertension, IL-17A together with IFN-γ interferes with the pressure natriuretic decrease in proximal tubule sodium transporters [90]. IL-17A^−/−^ abolished the activation of distal tubule transporters, specifically the sodium chloride co-transporter and the epithelial sodium channel and protected mice from glomerular and tubular injury [91]. Interestingly, the single dose of Ang II initiated neuronal and immune cell activity and affected circulating levels of CD4+IL-17+ T cells and increased IL-17 levels in WKY rats [92]. These changes are essential in the developing of hypertensive phenotype in WKY rats [92]. Recently, Wang and colleagues have shown that the deficiency in P-selectin glycoprotein ligand-1 was associated with reduced blood pressure upon Ang II infusion. Interestingly, this effect resulted in reduced plasma IL-17 level in Psgl-1^−/−^ mice compared with wild type (WT) animals. The administration of recombinant IL-17 restored the reduced response to Ang II [93]. IL-17 expression is regulated by Tri-partite motif (TRIM) 21 [94]. The lack of TRIM21in Ldlr^−/−^ mice resulted in elevated CD4+ T cells in the periphery and increased IL-17 mRNA expression in plaque suggesting that this molecule is a regulator of tissue inflammation and pro-inflammatory cytokine production [94].

In humans, the serum level of IL-17 was significantly increased in hypertensive subjects compared with normotensive individuals [86•]. Yao et al. have further confirmed that serum concentration of IL-17 was significantly increased in prehypertensive subjects in comparison with optimal blood pressure individuals and elevated level of this pro-inflammatory cytokine was accompanied by a rise in systolic blood pressure [95]. Especially, CD4+ T cells of humans with hypertension produced higher amounts of IL-17A than normotensive controls [3].

Interestingly, prolonged hypertension influences IL-17A serum levels and anti-hypertensive diuretic treatment was associated with higher IL-17A concentrations suggesting that arterial hypertension stimulates immune response independently of the blood pressure regulation [96]. Other well defined adaptive immunity cytokines in hypertension include IFN-γ and TNF-α. In the experimental model of hypertension and in human hypertensive individuals, the pivotal role of IFN-γ was confirmed. Marko et al. have demonstrated that IFN-γ plays an important role in experimental Ang II–dependent hypertension. IFN-γR^−/−^ mice had reduced cardiac hypertrophy and reduced cardiac infiltration of both macrophage and T cell and less fibrosis in comparison with WT upon Ang II administration [97]. IFN-γR deficiency was associated with reduced inflammation and improved glomerular infiltration rate in the kidney and increased albuminuria in comparison with the control animals [97]. IFN-γ deficiency resulted in blunted hypertension in response to Ang II infusion. Increased number of IFN-γ+CD8+ T cells in the spleen and kidneys of Lnk^−/−^ mice compared with WT mice was observed upon Ang II administration [36]. Our results confirmed that IFN-γ plays an important role in experimental hypertension. Angiotensin II increased the expression of mRNA encoding IFN-γ in perivascular adipose tissue [2]. A small number of CD8+ T cells produced this cytokine at the baseline and chronic Ang II infusion increased the content of IFN-γ+CD8+ lymphocytes in perivascular adipose tissue [2, 41]. Double-negative T cells produced significant amounts of IFN-γ in Ang II–infused mice, but this production was lower in comparison with CD8+ T cells. Interestingly, we observed that the incubation of the aorta with IFN-γ caused endothelial dysfunction. This effect was partially reversed by pre-incubation of the vessel with PEG-SOD [2]. CD8+ T cells produced higher amounts of IFN-γ in patients with hypertension in comparison with normotensive controls [3].

The hypertensive responses are also aggravated by TNF. Despite that TNF-α inhibitor etanerecept had no effect on arterial blood pressure in DOCA-salt hypertensive rats, it lowered both the proteinuria and cortical NF*-*κB activity [98]. Interestingly, using TNF-α^−/−^ mice, Sriramula et al. showed that Ang II–induced increase in arterial pressure in WT mice was absent in knockout animals. Also, cardiac hypertrophy was attenuated in TNF-α^−/−^ mice and the therapy with recombinant TNF restored all responses induced by Ang II in WT [99]. TNF^−/−^ mice had blunted hypertensive responses and reduced end-organ damage in a model of chronic kidney disease. This effect was associated with augmented eNOS expression in the kidney and enhanced NO bioavailability in TNF-lacking animals [100]. TNF-alpha in the kidney contributed to the development of hypertension and renal injury in Dahl salt-sensitive (SS) rats [101]. Intrarenal TNF-α increased by high-salt diet in Dahl SS rats, and etanerecept administration improved renal damage and hypertension [101]. In rats with renovascular hypertension, the injection of TNFR1 neutralizing antibody returned blood pressure to normotensive level. Interestingly, TNF-alpha administration into normotensive rats increased blood pressure and sympathetic nerve activity predominantly to the heart. TNFR1 neutralizing antibody abolished this effect [102]. Recently, we have shown that the percentage of both CD4+TNF+ and CD8+TNF+ T cells in perivascular adipose tissue was higher in Ang II–dependent hypertension in comparison with control animals. This was also true for TNF+CD3+CD4−CD8− T cells [41]. Anti-inflammatory interventions decreased the percentage of TNF+CD8+ T cells and did not affect other examined subsets [41].

While the pro-inflammatory cytokines such as IL-17, IFN-γ, and TNF-α have a detrimental effect in the pathogenesis of hypertension, the role of anti-inflammatory IL-10 is protective. Didion et al. have demonstrated that the systemic administration of Ang II resulted in marked impairment of endothelial function in IL-10^−/−^ mice in comparison with WT despite the similar increases in arterial blood pressure. Endogenous IL-10 limited Ang II–mediated oxidative stress and superoxide scavenging in IL-10^−/−^ mice improved vascular function [103]. Moreover, the treatment of hypertensive mice with IL-10 reduced both systolic blood pressure and activity of NADPH oxidases. Also, improved endothelial function in mesenteric resistance artery was observed in comparison with untreated hypertensive animals [104]. IL-10 infusion prevented the blood pressure increase and vascular dysfunction in Ang II–infused WT mice. This effect was associated with the modulatory role of IL-10 to limit of RhoA/Rho-kinase signaling pathway [105]. Barhoumi at el. provided evidence that hypertension is associated with reduced T regulatory cells numbers [106•]. The adoptive transfer of CD4+CD25+ Treg subsets but not CD4+CD25− T effector cells suppressed Ang II–mediated vascular injury in part through anti-inflammatory actions [106•]. Another study performed by Matrougui et al. revealed that Tregs are essential in the development of coronary arteriolar endothelial dysfunction in hypertension. The injection of Tregs from control mice to hypertensive animals reduced TNF-α release and improved endothelium-dependent relaxation [107]. The transfer of Tregs caused improved cardiac hypertrophy and less cardiac fibrosis but did not prevent an increase in blood pressure in mice. In this study, the reduction of CD4+, CD8+, and CD69+ cells and macrophage in the heart was noticed [108]. Also, in experimental preeclampsia, IL-10 administration normalized blood pressure and endothelial function. This effect was mediated by decreased plasma levels of endothelin-1, circulating and placental IFN-γ, and aortic and placental PECAM [109]. Anti-hypertensive effect of IL-10, in both the thoracic aorta and VSMC manifested in decreased systolic blood pressure in SHR, is mediated by CCL5 [110]. Interestingly, the serum levels of IL-10 and FoxP3 mRNA expression and the percentage of Treg (CD4+CD25+) were decreased in pregnancy-induced hypertensive (PIH) patients. At the same time, a significant increase of PD-1 in Treg was found in PIH compared with normal pregnancy. The PD-ligand 1 Fc increased Treg number and elevated TGF-β and IL-10 expression. The blocking of PD-L1 by using monoclonal antibody reversed this effect [111]. The suppression of Treg can be modulated by Nox2 [112]. Nox2 deficiency increased the number of Treg in the heart at the baseline, and Ang II inhibited the infiltration of effector T cells (Teff). Mice with Nox2 deficiency in CD4+ T cells showed inhibition of Ang II–induced hypertension and cardiac remodeling due to increased Treg and reduction in Th17 content. This protective effect was reversed by anti-CD25 antibody [112].

In Ang II–dependent hypertension, there is an imbalance between Th17/Treg in the spleen and in renal/cardiac infiltrating lymphocytes resulting in increased expressions of IL-17A, IL-23, and TNF-α, and decreased expression of IL-10. This imbalance in Th17/Treg in Ang II–induced hypertension is caused by serum/glucocorticoid-regulated kinase 1 (SGK1) [113]. Using of EMD638683-SGK1 inhibitor reversed cardiac and renal dysfunction induced by Ang II [113]. Also, the mechanism of the microvascular dysfunction in mice with established hypertension is associated with depletion in Treg [114]. In hypertensive mice, Treg displayed enhanced apoptosis and increased autophagy in mesenteric artery. Interestingly, the inhibition of autophagy or transfer of Treg into mice with establishes hypertension improved microvascular function [114].

## Conclusion

Adaptive immune responses, both T cell and B cell mediated, play a pivotal role in the development of hypertension and in mediating target organ damage. Adaptive immunity activation of both T cells and B cells is initiated early in the course of the disease and greatly contributes to important pathogenetic changes, through release of pro-inflammatory cytokines and antibodies. This leads to changes of renal sodium transporter expression and vascular endothelial as well as cardiac, renal, and perivascular fibrosis. While these mechanisms have been well defined in animal models, less evidence is available in humans. Unquestionably, such a large body of evidence warrants the development of novel anti-hypertensive strategies targeting adaptive immunity hypertensive mechanisms.

## References

[1] Guzik TJ, Hoch NE, Brown KA (2007). Role of the T cell in the genesis of angiotensin II induced hypertension and vascular dysfunction. J Exp Med.

[2] Mikolajczyk TP, Nosalski R, Szczepaniak P (2016). Role of chemokine RANTES in the regulation of perivascular inflammation, T-cell accumulation, and vascular dysfunction in hypertension. FASEB J.

[3] Itani HA, McMaster WG, Saleh MA (2016). Activation of human T cells in hypertension: studies of humanized mice and hypertensive humans. Hypertension..

[4] Loperena R, Van Beusecum JP, Itani HA (2018). Hypertension and increased endothelial mechanical stretch promote monocyte differentiation and activation: roles of STAT3, interleukin 6 and hydrogen peroxide. Cardiovasc Res.

[5] Ye J, Que B, Huang Y (2019). Interleukin-12p35 knockout promotes macrophage differentiation, aggravates vascular dysfunction and elevates blood pressure in angiotensin II-infused mice. Cardiovasc Res.

[6] Jansen T, Kroller-Schon S, Schonfelder T (2018). alpha1AMPK deletion in myelomonocytic cells induces a pro-inflammatory phenotype and enhances angiotensin II-induced vascular dysfunction. Cardiovasc Res.

[7] Guzik TJ, Skiba DS, Touyz RM, Harrison DG (2017). The role of infiltrating immune cells in dysfunctional adipose tissue. Cardiovasc Res.

[8] Norlander AE, Madhur MS, Harrison DG (2018). The immunology of hypertension. J Exp Med.

[9] •• Drummond GR, Vinh A, Guzik TJ, Sobey CG. Immune mechanisms of hypertension. Nat Rev Immunol. 2019. **State-of-the-art review of innate and adaptive immunity mechanisms of hypertension.** 10.1038/s41577-019-0160-5 30992524

[10] Krishnan SM, Dowling JK, Ling YH (2016). Inflammasome activity is essential for one kidney/deoxycorticosterone acetate/salt-induced hypertension in mice. Br J Pharmacol.

[11] Madhur MS, Harrison DG (2013). Senescent T cells and hypertension: new ideas about old cells. Hypertension..

[12] Hevia D, Araos P, Prado C (2018). Myeloid CD11c(+) antigen-presenting cells ablation prevents hypertension in response to angiotensin II plus high-salt diet. Hypertension..

[13] Kirabo A, Fontana V, de Faria AP (2014). DC isoketal-modified proteins activate T cells and promote hypertension. J Clin Invest.

[14] Dai X, Huang S, He Z (2017). Dysfunction of the thymus in mice with hypertension. Exp Ther Med.

[15] Bu DX, Lichtman AH (2010). T cells and blood vessels: costimulation turns up the pressure. Circulation..

[16] Barski A, Cuddapah S, Kartashov AV (2017). Rapid recall ability of memory T cells is encoded in their epigenome. Sci Rep.

[17] Itani HA, Xiao L, Saleh MA (2016). CD70 exacerbates blood pressure elevation and renal damage in response to repeated hypertensive stimuli. Circ Res.

[18] Takeichi N, Suzuki K, Okayasu T, Kobayashi H (1980). Immunological depression in spontaneously hypertensive rats. Clin Exp Immunol.

[19] Takeichi N, Suzuki K, Kobayashi H (1981). Characterization of immunological depression in spontaneously hypertensive rats. Eur J Immunol.

[20] Ba D, Takeichi N, Kodama T, Kobayashi H (1982). Restoration of T cell depression and suppression of blood pressure in spontaneously hypertensive rats (SHR) by thymus grafts or thymus extracts. J Immunol.

[21] Fannon LD, Braylan RC, Phillips MI (1992). Alterations of lymphocyte populations during development in the spontaneously hypertensive rat. J Hypertens.

[22] Rodriguez-Iturbe B, Quiroz Y, Ferrebuz A, Parra G, Vaziri ND (2004). Evolution of renal interstitial inflammation and NF-kappaB activation in spontaneously hypertensive rats. Am J Nephrol.

[23] Tipton AJ, Baban B, Sullivan JC (2012). Female spontaneously hypertensive rats have greater renal anti-inflammatory T lymphocyte infiltration than males. Am J Phys Regul Integr Comp Phys.

[24] Bell R, Herring SM, Gokul N (2011). High-resolution identity by descent mapping uncovers the genetic basis for blood pressure differences between spontaneously hypertensive rat lines. Circ Cardiovasc Genet.

[25] Braun MC, Herring SM, Gokul N (2014). Hypertensive renal injury is associated with gene variation affecting immune signaling. Circ Cardiovasc Genet.

[26] Gonzalez-Garay ML, Cranford SM, Braun MC, Doris PA (2014). Diversity in the preimmune immunoglobulin repertoire of SHR lines susceptible and resistant to end-organ injury. Genes Immun.

[27] Rodriguez-Iturbe B, Quiroz Y, Nava M (2002). Reduction of renal immune cell infiltration results in blood pressure control in genetically hypertensive rats. Am J Physiol Ren Physiol.

[28] Rodriguez-Iturbe B, Pons H, Quiroz Y (2001). Mycophenolate mofetil prevents salt-sensitive hypertension resulting from angiotensin II exposure. Kidney Int.

[29] De Miguel C, Das S, Lund H, Mattson DL (2010). T lymphocytes mediate hypertension and kidney damage in Dahl salt-sensitive rats. Am J Phys Regul Integr Comp Phys.

[30] De Miguel C, Lund H, Mattson DL (2011). High dietary protein exacerbates hypertension and renal damage in Dahl SS rats by increasing infiltrating immune cells in the kidney. Hypertension..

[31] Viel EC, Lemarie CA, Benkirane K, Paradis P, Schiffrin EL (2010). Immune regulation and vascular inflammation in genetic hypertension. Am J Physiol Heart Circ Physiol.

[32] Mattson DL, Lund H, Guo C, Rudemiller N, Geurts AM, Jacob H (2013). Genetic mutation of recombination activating gene 1 in Dahl salt-sensitive rats attenuates hypertension and renal damage. Am J Phys Regul Integr Comp Phys.

[33] Crowley SD, Song YS, Lin EE, Griffiths R, Kim HS, Ruiz P (2010). Lymphocyte responses exacerbate angiotensin II-dependent hypertension. Am J Phys Regul Integr Comp Phys.

[34] Shao J, Nangaku M, Miyata T (2003). Imbalance of T-cell subsets in angiotensin II-infused hypertensive rats with kidney injury. Hypertension..

[35] Trott DW, Thabet SR, Kirabo A (2014). Oligoclonal CD8+ T cells play a critical role in the development of hypertension. Hypertension..

[36] Saleh MA, McMaster WG, Wu J (2015). Lymphocyte adaptor protein LNK deficiency exacerbates hypertension and end-organ inflammation. J Clin Invest.

[37] Marvar PJ, Thabet SR, Guzik TJ (2010). Central and peripheral mechanisms of T-lymphocyte activation and vascular inflammation produced by angiotensin II-induced hypertension. Circ Res.

[38] Carnevale D, Pallante F, Fardella V (2014). The angiogenic factor PlGF mediates a neuroimmune interaction in the spleen to allow the onset of hypertension. Immunity.

[39] Perrotta M, Lori A, Carnevale L (2018). Deoxycorticosterone acetate-salt hypertension activates placental growth factor in the spleen to couple sympathetic drive and immune system activation. Cardiovasc Res.

[40] Carnevale L, D’Angelosante V, Landolfi A (2018). Brain MRI fiber-tracking reveals white matter alterations in hypertensive patients without damage at conventional neuroimaging. Cardiovasc Res.

[41] Mikolajczyk TP, Nosalski R, Skiba DS, et al. 1,2,3,4,6-Penta-O-galloyl-beta-d-glucose modulates perivascular inflammation and prevents vascular dysfunction in angiotensin II-induced hypertension. Br J Pharmacol. 2019. 10.1111/bph.14583 PMC6534792 30658013

[42] Sorop O, Heinonen I, van Kranenburg M (2018). Multiple common comorbidities produce left ventricular diastolic dysfunction associated with coronary microvascular dysfunction, oxidative stress, and myocardial stiffening. Cardiovasc Res.

[43] Touyz RM, Alves-Lopes R, Rios FJ (2018). Vascular smooth muscle contraction in hypertension. Cardiovasc Res.

[44] Stenmark KR, Frid MG, Graham BB, Tuder RM (2018). Dynamic and diverse changes in the functional properties of vascular smooth muscle cells in pulmonary hypertension. Cardiovasc Res.

[45] Vinh A, Chen W, Blinder Y (2010). Inhibition and genetic ablation of the B7/CD28 T-cell costimulation axis prevents experimental hypertension. Circulation.

[46] Shah KH, Shi P, Giani JF (2015). Myeloid suppressor cells accumulate and regulate blood pressure in hypertension. Circ Res.

[47] Siedlinski M, Nosalski R, Szczepaniak P (2017). Vascular transcriptome profiling identifies sphingosine kinase 1 as a modulator of angiotensin II-induced vascular dysfunction. Sci Rep.

[48] Youn JC, Yu HT, Lim BJ (2013). Immunosenescent CD8+ T cells and C-X-C chemokine receptor type 3 chemokines are increased in human hypertension. Hypertension..

[49] Tipton AJ, Sullivan JC (2014). Sex differences in T cells in hypertension. Clin Ther.

[50] Ji H, Pesce C, Zheng W (2005). Sex differences in renal injury and nitric oxide production in renal wrap hypertension. Am J Physiol Heart Circ Physiol.

[51] Sullivan JC, Semprun-Prieto L, Boesen EI, Pollock DM, Pollock JS (2007). Sex and sex hormones influence the development of albuminuria and renal macrophage infiltration in spontaneously hypertensive rats. Am J Phys Regul Integr Comp Phys.

[52] Crowson CS, Liao KP, Davis JM (2013). Rheumatoid arthritis and cardiovascular disease. Am Heart J.

[53] Sinicato NA, da Silva Cardoso PA, Appenzeller S (2013). Risk factors in cardiovascular disease in systemic lupus erythematosus. Curr Cardiol Rev.

[54] Mikolajczyk TP, Osmenda G, Batko B (2016). Heterogeneity of peripheral blood monocytes, endothelial dysfunction and subclinical atherosclerosis in patients with systemic lupus erythematosus. Lupus..

[55] Laresgoiti-Servitje E (2013). A leading role for the immune system in the pathophysiology of preeclampsia. J Leukoc Biol.

[56] Cornelius DC, Hogg JP, Scott J (2013). Administration of interleukin-17 soluble receptor C suppresses TH17 cells, oxidative stress, and hypertension in response to placental ischemia during pregnancy. Hypertension..

[57] Tipton AJ, Baban B, Sullivan JC (2014). Female spontaneously hypertensive rats have a compensatory increase in renal regulatory T cells in response to elevations in blood pressure. Hypertension.

[58] Brinson KN, Elmarakby AA, Tipton AJ (2013). Female SHR have greater blood pressure sensitivity and renal T cell infiltration following chronic NOS inhibition than males. Am J Phys Regul Integr Comp Phys.

[59] Pettersson US, Walden TB, Carlsson PO, Jansson L, Phillipson M (2012). Female mice are protected against high-fat diet induced metabolic syndrome and increase the regulatory T cell population in adipose tissue. PLoS One.

[60] Ji H, Zheng W, Li X (2014). Sex-specific T-cell regulation of angiotensin II-dependent hypertension. Hypertension..

[61] Pollow DP, Uhrlaub J, Romero-Aleshire M (2014). Sex differences in T-lymphocyte tissue infiltration and development of angiotensin II hypertension. Hypertension..

[62] Sandberg K, Ji H, Hay M (2015). Sex-specific immune modulation of primary hypertension. Cell Immunol.

[63] Xue B, Zhao Y, Johnson AK, Hay M (2008). Central estrogen inhibition of angiotensin II-induced hypertension in male mice and the role of reactive oxygen species. Am J Physiol Heart Circ Physiol.

[64] Xue B, Singh M, Guo F, Hay M, Johnson AK (2009). Protective actions of estrogen on angiotensin II-induced hypertension: role of central nitric oxide. Am J Physiol Heart Circ Physiol.

[65] Sullivan JC, Gillis EE (2017). Sex and gender differences in hypertensive kidney injury. Am J Physiol Ren Physiol.

[66] Schneider-Hohendorf T, Gorlich D, Savola P (2018). Sex bias in MHC I-associated shaping of the adaptive immune system. Proc Natl Acad Sci U S A.

[67] Chan CT, Sobey CG, Lieu M (2015). Obligatory role for B cells in the development of angiotensin II-dependent hypertension. Hypertension..

[68] Dingwell LS, Shikatani EA, Besla R (2019). B-cell deficiency lowers blood pressure in mice. Hypertension..

[69] Wu T, Ma J, Chen S (2001). Association of plasma antibodies against the inducible Hsp70 with hypertension and harsh working conditions. Cell Stress Chaperones.

[70] Pockley AG, De Faire U, Kiessling R, Lemne C, Thulin T, Frostegard J (2002). Circulating heat shock protein and heat shock protein antibody levels in established hypertension. J Hypertens.

[71] Zhang X, He MA, Cheng L (2008). Joint effects of antibody to heat shock protein 60, hypertension, and diabetes on risk of coronary heart disease in Chinese. Clin Chem.

[72] Wallukat G, Homuth V, Fischer T (1999). Patients with preeclampsia develop agonistic autoantibodies against the angiotensin AT1 receptor. J Clin Invest.

[73] Siddiqui AH, Irani RA, Blackwell SC, Ramin SM, Kellems RE, Xia Y (2010). Angiotensin receptor agonistic autoantibody is highly prevalent in preeclampsia: correlation with disease severity. Hypertension..

[74] Fu ML, Herlitz H, Schulze W (2000). Autoantibodies against the angiotensin receptor (AT1) in patients with hypertension. J Hypertens.

[75] Zhu F, Sun Y, Wang M (2011). Correlation between HLA-DRB1, HLA-DQB1 polymorphism and autoantibodies against angiotensin AT(1) receptors in Chinese patients with essential hypertension. Clin Cardiol.

[76] Liao YH, Wei YM, Wang M, Wang ZH, Yuan HT, Cheng LX (2002). Autoantibodies against AT1-receptor and alpha1-adrenergic receptor in patients with hypertension. Hypertens Res.

[77] Luther HP, Homuth V, Wallukat G (1997). Alpha 1-adrenergic receptor antibodies in patients with primary hypertension. Hypertension..

[78] Fu ML, Herlitz H, Wallukat G (1994). Functional autoimmune epitope on alpha 1-adrenergic receptors in patients with malignant hypertension. Lancet..

[79] Wenzel K, Haase H, Wallukat G (2008). Potential relevance of alpha(1)-adrenergic receptor autoantibodies in refractory hypertension. PLoS One.

[80] Wallukat G, Blasig IE, Morwinski R, Herrmann HJ, Rohde E (1995). The sera of spontaneously hypertensive rats contain agonistic auto-antibodies against the beta 1-adrenoceptor. J Hypertens.

[81] Ofosu-Appiah W, Huang LY, Kuhnle M, Sfeir G, Kennel A (1996). Autoantibodies against arterial antigens: characterization by ELISA and immunoblot analysis in the spontaneously hypertensive rat. Clin Exp Hypertens.

[82] Jahns R, Boivin V, Hein L (2004). Direct evidence for a beta 1-adrenergic receptor-directed autoimmune attack as a cause of idiopathic dilated cardiomyopathy. J Clin Invest.

[83] Zhou ZH, Wang J, Xiao H (2008). A novel autoantibody in patients with primary hypertension: antibody against L-type Ca2+ channel. Chin Med J.

[84] Amador CA, Barrientos V, Pena J (2014). Spironolactone decreases DOCA-salt-induced organ damage by blocking the activation of T helper 17 and the downregulation of regulatory T lymphocytes. Hypertension..

[85] Nguyen H, Chiasson VL, Chatterjee P, Kopriva SE, Young KJ, Mitchell BM (2013). Interleukin-17 causes Rho-kinase-mediated endothelial dysfunction and hypertension. Cardiovasc Res.

[86] Madhur MS, Lob HE, McCann LA (2010). Interleukin 17 promotes angiotensin II-induced hypertension and vascular dysfunction. Hypertension.

[87] Kleinewietfeld M, Manzel A, Titze J (2013). Sodium chloride drives autoimmune disease by the induction of pathogenic TH17 cells. Nature..

[88] Wu C, Yosef N, Thalhamer T (2013). Induction of pathogenic TH17 cells by inducible salt-sensing kinase SGK1. Nature.

[89] Saleh MA, Norlander AE, Madhur MS (2016). Inhibition of interleukin 17-a but not interleukin-17F signaling lowers blood pressure and reduces end-organ inflammation in angiotensin II-induced hypertension. JACC Basic Transl Sci.

[90] Kamat NV, Thabet SR, Xiao L (2015). Renal transporter activation during angiotensin-II hypertension is blunted in interferon-gamma−/− and interleukin-17A−/− mice. Hypertension..

[91] Norlander AE, Saleh MA, Kamat NV (2016). Interleukin-17A regulates renal sodium transporters and renal injury in angiotensin II-induced hypertension. Hypertension..

[92] Zubcevic J, Santisteban MM, Perez PD (2017). A single angiotensin II hypertensive stimulus is associated with prolonged neuronal and immune system activation in Wistar-Kyoto rats. Front Physiol.

[93] Wang Q, Wang H, Wang J (2018). Angiotensin II-induced hypertension is reduced by deficiency of P-selectin glycoprotein ligand-1. Sci Rep.

[94] Brauner S, Jiang X, Thorlacius GE (2018). Augmented Th17 differentiation in Trim21 deficiency promotes a stable phenotype of atherosclerotic plaques with high collagen content. Cardiovasc Res.

[95] Yao W, Sun Y, Wang X, Niu K (2015). Elevated serum level of interleukin 17 in a population with prehypertension. J Clin Hypertens (Greenwich).

[96] Simundic T, Jelakovic B, Dzumhur A (2017). Interleukin 17A and toll-like receptor 4 in patients with arterial hypertension. Kidney Blood Press Res.

[97] Marko L, Kvakan H, Park JK (2012). Interferon-gamma signaling inhibition ameliorates angiotensin II-induced cardiac damage. Hypertension..

[98] Elmarakby AA, Quigley JE, Imig JD, Pollock JS, Pollock DM (2008). TNF-alpha inhibition reduces renal injury in DOCA-salt hypertensive rats. Am J Phys Regul Integr Comp Phys.

[99] Sriramula S, Haque M, Majid DS, Francis J (2008). Involvement of tumor necrosis factor-alpha in angiotensin II-mediated effects on salt appetite, hypertension, and cardiac hypertrophy. Hypertension..

[100] Zhang J, Patel MB, Griffiths R (2014). Tumor necrosis factor-alpha produced in the kidney contributes to angiotensin II-dependent hypertension. Hypertension..

[101] Huang B, Cheng Y, Usa K (2016). Renal tumor necrosis factor alpha contributes to hypertension in dahl salt-sensitive rats. Sci Rep.

[102] Korim WS, Elsaafien K, Basser JR, Setiadi A, May CN, Yao ST (2018). In renovascular hypertension, TNF-alpha type-1 receptors in the area postrema mediate increases in cardiac and renal sympathetic nerve activity and blood pressure. Cardiovasc Res.

[103] Didion SP, Kinzenbaw DA, Schrader LI, Chu Y, Faraci FM (2009). Endogenous interleukin-10 inhibits angiotensin II-induced vascular dysfunction. Hypertension..

[104] Kassan M, Galan M, Partyka M, Trebak M, Matrougui K (2011). Interleukin-10 released by CD4(+)CD25(+) natural regulatory T cells improves microvascular endothelial function through inhibition of NADPH oxidase activity in hypertensive mice. Arterioscler Thromb Vasc Biol.

[105] Lima VV, Zemse SM, Chiao CW (2016). Interleukin-10 limits increased blood pressure and vascular RhoA/Rho-kinase signaling in angiotensin II-infused mice. Life Sci.

[106] Barhoumi T, Kasal DA, Li MW (2011). T regulatory lymphocytes prevent angiotensin II-induced hypertension and vascular injury. Hypertension.

[107] Matrougui K, Abd Elmageed Z, Kassan M (2011). Natural regulatory T cells control coronary arteriolar endothelial dysfunction in hypertensive mice. Am J Pathol.

[108] Kvakan H, Kleinewietfeld M, Qadri F (2009). Regulatory T cells ameliorate angiotensin II-induced cardiac damage. Circulation..

[109] Tinsley JH, South S, Chiasson VL, Mitchell BM (2010). Interleukin-10 reduces inflammation, endothelial dysfunction, and blood pressure in hypertensive pregnant rats. Am J Phys Regul Integr Comp Phys.

[110] Kim HY, Cha HJ, Kim HS (2015). CCL5 upregulates IL-10 expression and partially mediates the antihypertensive effects of IL-10 in the vascular smooth muscle cells of spontaneously hypertensive rats. Hypertens Res.

[111] Jiang L, Tang C, Gong Y (2018). PD-1/PD-L1 regulates Treg differentiation in pregnancy-induced hypertension. Braz J Med Biol Res.

[112] Emmerson A, Trevelin SC, Mongue-Din H (2018). Nox2 in regulatory T cells promotes angiotensin II-induced cardiovascular remodeling. J Clin Invest.

[113] Du YN, Tang XF, Xu L, Chen WD, Gao PJ, Han WQ (2018). SGK1-FoxO1 signaling pathway mediates Th17/Treg imbalance and target organ inflammation in angiotensin II-induced hypertension. Front Physiol.

[114] Radwan E, Mali V, Haddox S (2019). Treg cells depletion is a mechanism that drives microvascular dysfunction in mice with established hypertension. Biochim Biophys Acta Mol basis Dis.

